# Negative symptoms in schizophrenia: a study in a large clinical sample of patients using a novel automated method

**DOI:** 10.1136/bmjopen-2015-007619

**Published:** 2015-09-03

**Authors:** Rashmi Patel, Nishamali Jayatilleke, Matthew Broadbent, Chin-Kuo Chang, Nadia Foskett, Genevieve Gorrell, Richard D Hayes, Richard Jackson, Caroline Johnston, Hitesh Shetty, Angus Roberts, Philip McGuire, Robert Stewart

**Affiliations:** 1 Department of Psychosis Studies, King's College London, Institute of Psychiatry, Psychology & Neuroscience, London, UK; 2 Department of Psychological Medicine, King's College London, Institute of Psychiatry, Psychology & Neuroscience, London, UK; 3 South London and Maudsley NHS Foundation Trust, Biomedical Research Centre Nucleus, London, UK; 4 Roche Products Limited, Welwyn Garden City, UK; 5 Department of Computer Science, The University of Sheffield, Portobello, Sheffield, UK; 6 Social Developmental and Genetic Psychiatry Department, King's College London, Institute of Psychiatry, Psychology & Neuroscience, London, UK

**Keywords:** negative symptoms, schizophrenia, psychosis, natural language processing, electronic health records, text mining

## Abstract

**Objectives:**

To identify negative symptoms in the clinical records of a large sample of patients with schizophrenia using natural language processing and assess their relationship with clinical outcomes.

**Design:**

Observational study using an anonymised electronic health record case register.

**Setting:**

South London and Maudsley NHS Trust (SLaM), a large provider of inpatient and community mental healthcare in the UK.

**Participants:**

7678 patients with schizophrenia receiving care during 2011.

**Main outcome measures:**

Hospital admission, readmission and duration of admission.

**Results:**

10 different negative symptoms were ascertained with precision statistics above 0.80. 41% of patients had 2 or more negative symptoms. Negative symptoms were associated with younger age, male gender and single marital status, and with increased likelihood of hospital admission (OR 1.24, 95% CI 1.10 to 1.39), longer duration of admission (β-coefficient 20.5 days, 7.6–33.5), and increased likelihood of readmission following discharge (OR 1.58, 1.28 to 1.95).

**Conclusions:**

Negative symptoms were common and associated with adverse clinical outcomes, consistent with evidence that these symptoms account for much of the disability associated with schizophrenia. Natural language processing provides a means of conducting research in large representative samples of patients, using data recorded during routine clinical practice.

**Strengths and limitations of this study**

- This is the largest known study (over 7000 participants) to investigate the relationship of negative symptoms with clinical outcomes in people with schizophrenia. Our findings demonstrate that negative symptoms are present in a substantial number of people with schizophrenia and are associated with increased hospital admission, readmission and duration of inpatient stay.
- To our knowledge, this is the first published study to use an automated information extraction method to acquire data on negative symptoms from electronic health records. This approach permits rapid acquisition of negative symptom data which is representative of everyday clinical practice in secondary mental healthcare.
- Our findings are based on data recorded by clinicians delivering routine mental healthcare who were not specifically ascertaining negative symptoms. It is therefore possible that negative symptoms were not comprehensively documented in the electronic health records from which they were identified leading to an inaccurate estimate of their prevalence in the analysed sample.

## Introduction

Negative symptoms, which include amotivation, a flattening of emotional responses, a reduction in speech and activity, and social withdrawal,^1^ contribute to much of the disability associated with schizophrenia.^2^ These symptoms are also associated with poor psychosocial functioning^3^ and a reduced likelihood of remission.^4–9^ The aetiology and pathophysiology of negative symptoms are unknown, and there are no effective treatments.^10^ ^11^

A number of excellent rating scales have been developed to assess negative symptoms.^12–14^ However, these are relatively detailed, require a trained rater, and are not routinely applied in clinical practice. As a result, much of our knowledge of negative symptoms is derived from studies in relatively small samples of patients, who may have been selected for inclusion because they had particularly severe symptoms. The findings from these samples may not therefore be representative of negative symptoms in the overall population of patients with schizophrenia.

Clinical information is increasingly recorded electronically, facilitating access of rich clinical data, including presence or absence of symptoms,^15^ from routine medical records. In the present study, we used a novel information extraction tool to identify negative symptomatology in a large body of electronic records collected from individuals with schizophrenia.^16–18^ We then examined the relationship between negative symptoms and clinical outcomes. We tested the hypothesis that negative symptoms are common in patients with schizophrenia, and are associated with poor clinical outcome, as indexed by the frequency and duration of hospital admissions.

## Methods

### Participants and clinical data

The study was carried out using the South London and Maudsley NHS Foundation Trust (SLaM) Biomedical Research Centre (BRC) Case Register, comprising electronic health record data dating back to April 2006 from a large mental healthcare provider to 1.2 million residents of southeast London (UK). The data were interrogated using the Clinical Record Interactive Search (CRIS) application,^19^ with a robust anonymisation process and patient-led oversight.^20^ Three samples were identified for analysis:

- Sample A (n=7678): patients with schizophrenia (International Classification of Diseases (ICD)-10 F20.XX) aged 16 years and over who had used SLaM services during 2011. This sample was used to investigate: (1) the relationship between negative symptoms, documented at any point in the electronic health record, and demographic and other clinical measures (described below); (2) the relationship between negative symptoms documented prior to 1 January 2011 and the risk of hospital admission during 2011. This year was chosen for analysis because it maximised the duration of time over which text would be available for measurement development, while allowing at least 12 months follow-up in all instances.
- Sample B (n=1612): the subset of patients from sample A who had been discharged from SLaM inpatient care during 2011. This sample was used to investigate the relationship between negative symptoms documented prior to 2011 and the risk of readmission in the 12 months following discharge.
- Sample C (n=1609): the subset of patients from sample A who received SLaM inpatient care during 2011. This sample was used to investigate the relationship between negative symptoms documented prior to 2011 and the length of the first hospital admission during 2011.

### Measurement development

Natural language processing (NLP) information extraction allows structured information to be obtained from unstructured text records. We used NLP to detect statements in the correspondence fields of clinical records to determine references to prespecified negative symptoms. Full details of the NLP method are described in a previous paper.^16^ In summary, a putative training data set was selected which contained broad dictionary terms relevant to the negative symptoms of interest (described below). A detailed review of the training data set was undertaken by two psychiatrists (RP and RS) to identify and annotate key phrases within the records that were either relevant or irrelevant for keywords related to each symptom. Inter-rater reliability was tested between the two annotators resulting in percentage agreement of 93.0% (Cohen's κ 0.85). This training data set was used to construct an application (CRIS Negative Symptoms Scale, CRIS-NSS) using a hybrid classification model consisting of a support vector machine (SVM) learning algorithm^21^ and rule-based text matching, using the Generalised Architecture for Text Engineering (GATE) software package.^17^ The SVM algorithm was applied using a ‘bag-of-words’ approach to take into account the context of negative symptoms within the sentence in which they were documented, thereby allowing ascertainment of negative symptoms experienced specifically by the patient as well as distinguishing between positive instances and negated instances.^16^ Once developed, CRIS-NSS was subsequently used to determine the presence of negative symptoms within the clinical sample. The accuracy of CRIS-NSS was evaluated using precision and recall statistics which were generated through internal fivefold cross-validation:^21^ precision, representing the proportion of text instances identified by the tool which were found to be correct in terms of identifying the negative symptom of interest (equivalent to positive predictive value); and recall, measuring the proportion of text instances recording a given negative symptoms which were correctly identified as such by the tool (equivalent to sensitivity).

Details of the criteria for ascertaining the negative symptoms in the CRIS-NSS application are described in further detail elsewhere;^16^ briefly, applications were developed for 10 items: poor motivation, blunted or flattened affect, poor eye contact, emotional withdrawal, poor rapport, social withdrawal, poverty of speech, mutism, apathy and concrete thinking. Each of these symptoms was defined as a binary variable on the basis of being present at any point in the record within the defined time period, and a composite scale (range 0–10) was constructed by summing these variables, followed by Cronbach α score calculation (a measure of intercorrelation between individual scale items) to estimate its internal consistency. A threshold score of at least 2 (ie, two or more negative symptoms documented) was applied a priori to determine the presence or absence of negative symptoms for analysis as a binary variable, as well as treating the scale score as an ordinal variable.

### Clinical outcome measures and covariates

The following clinical and demographic variables were obtained as covariates from the data set: age (on 1 January 2011), gender, marital status, employment status, and admission and discharge dates for inpatient care episodes. Using structured data derived from the Health of the Nation Outcome Scale (HoNOS),^22^ routinely completed in SLaM patients, the following subscales (scored 0–4) were used as covariates: activities of daily living (ADL) impairment, problems with relationships (social impairment), presence of hallucinations or delusions (a measure of positive symptoms) and depressive symptoms. For all of these HoNOS subscales, binary variables were defined on the basis of a score of 2 or more indicating the presence of each construct at levels judged to be clinically significant. In cases with multiple data points, all covariates were defined as those recorded closest to 1 January 2011.

### Statistical analysis

STATA (V.11) software was used. Estimates of prevalence of negative symptoms by demographic factors were obtained as the proportion of patients within each group with two or more negative symptoms. After describing the distribution of negative symptoms and the psychometric properties of the CRIS-NSS, further analyses were performed to investigate the associations between the clinical outcomes described above and (1) the presence of negative symptoms, using binary logistic regression; and (2) CRIS-NSS scores, using ordinal logistic regression. Reference groups for categorical variables were generally defined as the most prevalent category, apart from age group where the youngest group of sufficient size was assigned as the reference. Associations between negative symptomatology and hospital admission and readmission were analysed using logistic regression, while those with length of inpatient stay were analysed using linear regression—again, estimating associations with both the binary and ordinal CRIS-NSS exposure. For the analyses with hospitalisation outcomes in/following 2011, CRIS-NSS was generated restricting information extraction to electronic health records prior to 2011. Where data were missing on individual covariates (in 2362 participants), this was indicated in the regression models as a separate category, supplemented by sensitivity analyses performed on the sample with complete data on all covariates to check the consistency of findings. A further supplementary analysis was performed to test the hypothesis that the association between negative symptoms and clinical outcomes varies with age. For this analysis, the previous analyses were repeated within the subgroups of those aged under the age of 40 years and those over the age of 40 years and including an interaction term of age under or over 40 and binary CRIS-NSS exposure. Finally, secondary analyses were undertaken to investigate and compare the relationships of individual CRIS-NSS symptoms with risk of readmission and length of stay using binary logistic and linear regression, respectively.

## Results

### Performance of CRIS-NSS

Table 1 illustrates results from fivefold cross-validation of the CRIS-NSS tool. Precision coefficients ranged between 0.80 and 0.99 and recall between 0.62 and 0.97. For the composite 10-point scale, the Cronbach α value was 0.78 indicating a good level of internal consistency.

**Table 1:** Performance of Clinical Record Interactive Search Negative Symptoms Scale (CRIS-NSS) information extraction applications ascertaining individual symptom domains

Symptom	Precision/ recall	Prevalence (%) in patients with schizophrenia receiving care during 2011 (n=7678)
Poor motivation	0.87/0.62	30.5
Blunted or flattened affect	0.93/0.83	27.4
Poor eye contact	0.95/0.79	26.0
Emotional withdrawal	0.85/0.74	23.5
Poor rapport	0.91/0.77	16.3
Social withdrawal	0.94/0.96	12.7
Poverty of speech	0.80/0.73	12.4
Mute	0.99/0.94	8.1
Apathy	0.88/0.97	7.7
Concrete thinking	0.91/0.72	5.7

### Prevalence and distribution of negative symptoms

Of the 7678 patients with schizophrenia, 3149 (41.0%) had at least two negative symptoms documented. Table 1 displays prevalences for each of the symptoms classified by the tool. The most frequently recorded symptoms were poor motivation (30.5%), blunted or flattened affect (27.4%), poor eye contact (26.0%) and emotional withdrawal (23.5%). The prevalences by number of symptoms were as follows: one symptom 14.6%, two symptoms 12.7%, three symptoms 9.3%, four symptoms 6.4%, five symptoms 5.0%, six or more symptoms 7.6%.

Binary logistic regression analyses (table 2) revealed that patients with two or more negative symptoms were most likely to be 20–29 years old, male and single. Two or more negative symptoms were also associated with ADL impairment, whereas patients who were employed were less likely to have negative symptoms compared with those unemployed. Ordinal logistic regression analysis (etable 1) revealed similar findings for CRIS-NSS score as an exposure, and sensitivity analyses limited to those with full data on all covariates (etable 2) were also consistent.

**Table 2:** Binary logistic regression analysis of factors associated with negative symptoms in patients with schizophrenia (n=7678)

Factor	Group	Number in sample	Prevalence of two or more negative symptoms (%)	Association with two or more negative symptoms: OR (95% CI), p value			
				Unadjusted		Adjusted model (n=7676)*	
Age (years)	16–19	203	27.6	0.35 (0.25 to 0.49)	<0.001	0.50 (0.35 to 0.71)	<0.001
	20–29	1337	52.0	Reference		Reference	
	30–39	1775	47.0	0.82 (0.71 to 0.94)	0.006	0.85 (0.73 to 0.99)	0.033
	40–49	1983	42.6	0.69 (0.60 to 0.79)	<0.001	0.71 (0.61 to 0.82)	<0.001
	50–59	1137	37.2	0.55 (0.47 to 0.64)	<0.001	0.56 (0.47 to 0.67)	<0.001
	60–69	654	29.1	0.38 (0.31 to 0.46)	<0.001	0.39 (0.31 to 0.48)	<0.001
	70+	589	18.0	0.20 (0.16 to 0.26)	<0.001	0.22 (0.17 to 0.28)	<0.001
Gender	Male	4592	45.3	Reference		Reference	
	Female	3084	34.7	0.64 (0.59 to 0.71)	<0.001	0.77 (0.70 to 0.85)	<0.001
Marital status (most recent)	Single	5795	44.6	Reference		Reference	
	Married/cohabiting	785	31.6	0.57 (0.49 to 0.67)	<0.001	0.76 (0.64 to 0.90)	0.002
	Divorced/separated	776	33.4	0.62 (0.53 to 0.73)	<0.001	0.85 (0.71 to 1.00)	0.054
	Widowed	208	21.2	0.33 (0.24 to 0.47)	<0.001	0.77 (0.53 to 1.12)	0.178
Employment (most recent)	Unemployed	4956	47.9	Reference		Reference	
	Employed	341	39.6	0.71 (0.57 to 0.89)	0.003	0.68 (0.54 to 0.86)	0.001
	In education	311	39.6	0.71 (0.56 to 0.90)	0.004	0.81 (0.63 to 1.03)	0.089
	Retired	7	14.3	0.18 (0.02 to 1.51)	0.114	0.40 (0.04 to 3.52)	0.405
ADL impairment	Absent	4700	41.9	Reference		Reference	
	Present	2283	46.3	1.20 (1.08 to 1.32)	<0.001	1.35 (1.21 to 1.52)	<0.001
Social impairment	Absent	4432	42.7	Reference		Reference	
	Present	2533	44.4	1.07 (0.97 to 1.18)	0.158	0.94 (0.84 to 1.05)	0.240
Delusions/hallucinations	Absent	3904	41.9	Reference		Reference	
	Present	3077	45.0	1.14 (1.03 to 1.25)	0.009	1.19 (1.07 to 1.31)	0.001
Depression	Absent	4976	45.2	Reference		Reference	
	Present	2014	38.8	0.77 (0.69 to 0.85)	<0.001	0.74 (0.66 to 0.82)	<0.001

*Results adjusted for all the factors reported in this table; two cases with no recorded data on gender were dropped.

ADL, activities of daily living.

### Hospital admission, length of stay and readmission

**Figure 1:**
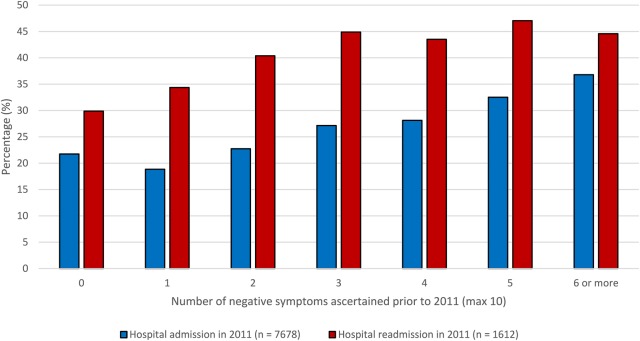
Percentage of patients admitted to hospital or readmitted to hospital following discharge in 2011 by number of negative symptoms.

**Figure 2:**
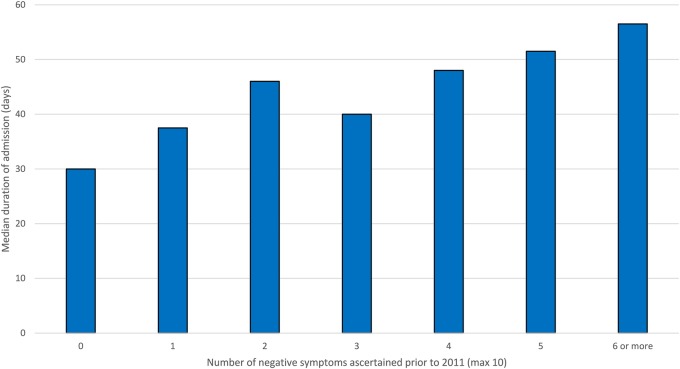
Median duration of admission among mental health inpatients with schizophrenia in 2011 by number of negative symptoms (n=1609).

Figure 1 summarises the association of negative symptoms recorded prior to 2011 with mental health admission (etable 3) and readmission (etable 4) in 2011. Figure 2 summarises length of hospitalisation for inpatients during 2011 (etable 5). Logistic and linear regression analyses (table 3) confirmed that negative symptoms were associated with a higher likelihood of admission, readmission and a longer duration of hospitalisation. Specifically, after full adjustment (table 3, model 3), patients with two or more negative symptoms before 2011 had a 24% greater likelihood of admission during 2011. Moreover, each of their admissions was, on average, an extra 21 days in duration, and when they were discharged, they had a 58% higher risk of readmission within 12 months. All of these associations remained independent and largely unaltered following adjustment for intensity of delusions/hallucinations among other covariates. Further analysis (etable 6) comparing patients aged under and over 40 years showed that the effects of negative symptoms on inpatient admission were broadly similar for both groups but with a slight increase in risk of readmission and reduced duration of admission in relation to negative symptoms for those under 40 compared with those over 40. However, the age × negative symptoms interaction term remained a non-significant factor (p>0.05) for all models.

**Table 3:** Association between number of negative symptoms ascertained prior to 2011 and mental health hospital admission, readmission and duration of admission in 2011

	Inpatient admission (OR, 95% CI; n=7678)*	Readmission within 12 months of inpatient admission (OR, 95% CI; n=1612)*	Duration of inpatient admission (days; β-coefficient, 95% CI; n=1609)†
Associations with 2 or more negative symptoms (binary variable)			
Unadjusted	1.47 (1.32 to 1.63)	1.73 (1.41 to 2.12)	23.9 (11.2 to 36.7)
1. Age and sex	1.37 (1.23 to 1.53)	1.70 (1.38 to 2.09)	24.1 (11.3 to 36.9)
2. Model 1 plus marital status and employment	1.27 (1.13 to 1.42)	1.58 (1.28 to 1.96)	20.1 (7.1 to 33.1)
3. Model 2 plus delusions/hallucinations, and depression	1.24 (1.10 to 1.39)	1.58 (1.28 to 1.95)	20.5 (7.6 to 33.5)
Associations with incremental number of negative symptoms (10-point scale ordinal variable)‡			
Unadjusted	1.12 (1.09 to 1.15)	1.12 (1.07 to 1.17)	6.5 (3.5 to 9.4)
1. Age and sex	1.09 (1.06 to 1.12)	1.11 (1.06 to 1.16)	6.3 (3.3 to 9.2)
2. Model 1 plus marital status and employment	1.07 (1.04 to 1.10)	1.09 (1.04 to 1.14)	5.4 (2.4 to 8.4)
3. Model 2 plus delusions/hallucinations, and depression	1.07 (1.04 to 1.10)	1.09 (1.04 to 1.14)	5.6 (2.6 to 8.6)

*Logistic regression.

†Linear regression.

‡ORs and β-coefficients are per one unit increase on the 10-point scale.

Finally, logistic and linear regression analyses were performed to examine the relationship between individual negative symptoms and the frequency and duration of admission (table 4). Poor eye contact and poor rapport were associated with increased risk of readmission, while apathy was associated with increased duration of admission. Emotional withdrawal and mutism were associated with both the risk of readmission and the duration of admission.

**Table 4:** Associations between individual Clinical Record Interactive Search Negative Symptoms Scale (CRIS-NSS) components and readmission risk/duration of admission in 2011

Negative symptom	Readmission risk (binary logistic regression) (n=1612)				Duration of admission (linear regression) (n=1590)			
	Unadjusted		Adjusted*		Unadjusted		Adjusted*	
	OR (95% CI)	p Value	OR (95% CI)	p Value	β-coefficient (95% CI)	p Value	β-coefficient (95% CI)	p Value
Poor motivation	1.40 (1.13 to 1.74)	0.002	1.29 (1.03 to 1.61)	0.026	23.0 (9.1 to 36.9)	0.001	19.1 (5.0 to 33.2)	0.008
Blunted or flattened affect	1.34 (1.08 to 1.65)	0.007	1.20 (0.97 to 1.50)	0.097	12.8 (−1.2 to 26.8)	0.073	8.3 (−5.7 to 22.4)	0.242
Poor eye contact	1.60 (1.30 to 1.98)	<0.001	1.48 (1.19 to 1.83)	<0.001	18.0 (4.2 to 31.8)	0.011	14.8 (0.9 to 28.6)	0.036
Emotional withdrawal	1.62 (1.30 to 2.02)	<0.001	1.49 (1.19 to 1.87)	0.001	32.5 (18.1 to 46.9)	<0.001	30.0 (15.6 to 44.4)	<0.001
Poor rapport	1.63 (1.29 to 2.06)	<0.001	1.50 (1.18 to 1.90)	0.001	23.1 (7.5 to 38.6)	0.004	21.1 (5.5 to 36.6)	0.008
Social withdrawal	1.16 (0.88 to 1.54)	0.291	1.02 (0.76 to 1.36)	0.911	16.4 (−2.9 to 35.7)	0.095	9.2 (−10.1 to 28.6)	0.349
Poverty of speech	1.30 (0.98 to 1.70)	0.064	1.12 (0.85 to 1.49)	0.416	13.2 (−5.8 to 32.2)	0.173	8.5 (−10.5 to 27.5)	0.379
Mute	1.71 (1.27 to 2.30)	<0.001	1.56 (1.15 to 2.12)	0.004	28.5 (7.9 to 49.1)	0.007	29.2 (8.6 to 49.7)	0.005
Apathy	1.02 (0.71 to 1.47)	0.914	0.93 (0.64 to 1.35)	0.692	32.5 (6.7 to 58.2)	0.013	27.4 (1.8 to 53.1)	0.036
Concrete thinking	1.37 (0.94 to 2.01)	0.100	1.25 (0.85 to 1.84)	0.250	16.8 (−10.2 to 43.9)	0.222	11.3 (−15.5 to 38.1)	0.407

*Adjusted for age, sex, marital status, employment status, presence of hallucinations/delusions and depression.

## Discussion

Using an SVM learning method with an NLP tool, we were able successfully to extract data on negative symptoms from the electronic mental health records of a large clinical sample of patients with schizophrenia. This approach did not require any specialised training or extra clinical assessments, and was able to generate a scale with robust construct and predictive validity from data recorded as part of routine clinical care.

The results suggest that negative symptoms are documented in the electronic health records of a sizeable proportion of patients with schizophrenia, particularly in those who are relatively young, male and not in a relationship, consistent with findings from studies that assessed negative symptoms using quite different methods.^23^ ^24^ Our findings were based on the unprompted documentation of negative symptoms in the context of routine clinical care by staff who were not specifically trained in their assessment. Previous findings have usually been based on systematic ratings by a researcher using a dedicated rating scale. Negative symptoms are relatively difficult to detect and assess,^1^ ^2^ and may be less frequently documented than positive symptoms, such as delusions and hallucinations, because they are less clinically obvious. In addition, mental health services in the UK are often orientated towards the management of acute crises, and hence the treatment of positive symptoms.^25^ It is thus possible that the figures for the prevalence and the severity of negative symptoms derived from our approach are lower than would have been obtained from a trained assessor using a standardised instrument. In addition, our method may be more likely to identify the types of negative symptoms (eg, poverty of speech) whose detection does not require specialised training.

We found that a substantial proportion (41%) of the sample had at least two negative symptoms. Although we defined and assessed negative symptoms in different ways to previous studies, this figure is comparable to that described in other samples of patients with schizophrenia (Jager *et al*^4^: 44%; Bobes *et al*^23^: 58%; Cohen *et al*^24^: 40%). Taken together, these findings suggest that negative symptoms are a relatively common feature of schizophrenia, rather than being limited to a subgroup of patients with a chronic, unremitting illness.^26^

As predicted, we found a clear association between negative symptoms and poor clinical outcomes, as indexed by impairments in daily living, increased risk of admission, increased duration of admission and increased risk of readmission. Hospital admissions are the main drivers of cost in the care of patients with schizophrenia,^27^ but have traditionally been linked to the severity of positive psychotic symptoms.^28^ Our data indicate that negative symptoms are an equally important factor, and suggest that a greater emphasis on assessing and treating these features of schizophrenia may have significant health economic benefits. However, as our findings are drawn from observational data, it would be necessary to perform interventional clinical studies to determine whether an effective treatment for negative symptoms would lead to better clinical outcomes.

A key strength of our study was the large size of the patient sample, and that it was representative of the overall clinical population of people with schizophrenia in a defined geographic area. Previous studies of negative symptoms have usually involved smaller patient samples that were recruited to a research project.^4^ ^23^ ^24^ Focusing the information extraction process on text from correspondence maximises the generalisability of our approach, as letters to primary care physicians (which accounted for a large portion of the correspondence text) are unlikely to vary substantially between mental health services with respect to the language used to describe the symptoms of interest. In the present study, we examined the patient's entire record rather than discrete periods of illness, and it was not possible to delineate the timing or duration of individual negative symptoms, or whether they were primary (ie, a direct consequence of illness) or secondary (eg, side effects of treatment) as these measures were not routinely documented in electronic health records. Although we investigated the association of negative symptoms in clinical documents prior to 1 January 2011 with outcomes occurring after 1 January 2011 (to ensure that negative symptoms were always ascertained prior to outcomes), if negative symptoms were identified prior to 1 January 2011, it was not possible to ascertain when they occurred prior to this date, or their temporal relationships to subsequent clinical outcomes. The findings were thus derived from assessments made over a period that was not standardised, but was generally relatively long. In contrast, most assessments of negative symptoms in the literature are derived from a single cross-sectional measurement.^29^ ^30^

A further limitation of our analysis was the extent to which individual negative symptoms could be considered as having equal weight in a composite score. Weighting the 10 negative symptom applications equally resulted in a composite score (from 0 to 10) with a reasonable degree of internal consistency, as demonstrated by a Cronbach α value of 0.78. However, analysing the association of each negative symptom with clinical outcomes revealed varying degrees of association with poor clinical outcomes for different negative symptoms. Future studies are necessary to examine the propensity for different negative symptoms to co-occur in individual patients and the extent to which different clusters of symptoms are associated with clinical outcomes, particularly in the light of previous research which suggests that negative symptoms segregate into two subdomains relating to amotivation and reduced emotional expression.^31^

The application of NLP to clinical records is unlikely to identify negative symptoms as accurately as a direct assessment using a specialised psychopathological rating scale. However, automated tools could be used to screen individuals and identify those with negative symptoms who would then benefit from comprehensive assessment using a standardised instrument. In this way, automated methods could be used to complement standardised instruments. Automated information extraction tools could also be developed to identify other clinical parameters from electronic health records in order to support real-time clinical decision-making. These possibilities could be explored in future research.

In summary, our data suggest that negative symptoms can be identified in clinical records using automated methods, are common in patients with schizophrenia and are associated with poor clinical outcomes. The findings highlight the potential of automated information extraction tools in mental health research and clinical practice, and the importance of developing effective treatments for negative symptoms.
